# HIV Infection and Acute Stroke: A Case Report and a Review of the Literature

**DOI:** 10.1155/2013/892054

**Published:** 2013-08-29

**Authors:** Hussein Assallum, Mohammad Alkayem, Nehad Shabarek

**Affiliations:** Department of Internal Medicine, Lincoln Medical and Mental Health Center, 234 E 149th Steet, Bronx, NY 10451, USA

## Abstract

*Background*. In the United States, ischemic stroke in HIV-infected patients has increased by 60%. However, unexpected cardiovascular events in relatively young patients have been observed. *Clinical Vignette.* A 31-year-old male who presented with a 5-hour history of sudden onset slurred speech and left hemiplegia. He has medical history of HIV infection for 2 years taking ARTs. On exam, a significant left hemiparesis was noticed. Brain MRI showed right anterior corona radiata and basal ganglia acute infarction. *Discussion.* Several mechanisms have been proposed for the relationship between HIV infection and cardiovascular risk. (i) HIV-associated dyslipidemia: HIV-infected patients tend to develop decrease in HDL-c and LDL-c levels. ART was associated with an increase in LDL-c but little change in HDL-c. (ii) Endothelial dysfunction: certain antiretroviral agents may independently contribute to endothelial damage. (iii) Hypertension: systolic blood pressure is higher in those using ART for greater than five years. (iv) Insulin resistance and diabetes have been noticed with ART. (v) Chronic inflammation. (vi) Hypercoagulability: decrease in proteins C and S was associated with HIV infection. *Conclusion.* Poorly controlled HIV infection and/or the introduction of ATR might be risk factors for cardiovascular events. More studies needed to address this medical dilemma.

## 1. Background

In the United States, stroke risk in human immunodeficiency virus- (HIV-) infected patients increased 60% over the decade from 1997 through 2006 [1]. Since the emergence of AIDS in 1981, substantial advances in our understanding of the acquired immune deficiency syndrome (AIDS) have been achieved.

In addition, therapy was dramatically altered with the introduction of antiretroviral drugs in 1987 and revolutionized by combination treatment, known as antiretroviral therapies (ARTs) in 1996 which dramatically decreased mortality and morbidity of HIV as EuroSIDA study had shown [2]. But soon after the introduction of protease inhibitors (PIs) and nonnucleoside reverse-transcriptase inhibitors (NNRTIs) for the management of HIV infection, clinicians observed unexpected cardiovascular events among patients receiving this new combination in patients who were relatively young. This concern was validated by several reports; the French Hospital Database on HIV found that patients who had been treated with protease inhibitor for 18 months or more had twice the risk of myocardial infarction that was seen among patients with less drug exposure [3]. A cross-sectional study carried out on HIV-infected patients had shown that stroke in patients with HIV/AIDS was not associated with opportunistic infections and tumors, and early assessment of the vascular status was recommended [4]. We are presenting the case of HIV-infected young patient with acute stroke to discuss the risk factors of cardiovascular events in patients with HIV infection on ART as stroke is a rising cause of disability in HIV and AIDS populations.

## 2. Epidemiology

In a cohort study comparing stroke incidence in HIV-infected patients versus non-HIV-infected patients, the incidence rate was 5.27 per 1000 person-years in HIV-infected patients compared with 3.75 in non-HIV-infected patients, with an unadjusted HR of 1.40 (95% confidence interval (CI): 1.17 to 1.69, *P* < 0.001) [5]. Another cohort study done by Vinikoor et al. in the University of North Carolina of a total of 2,515 HIV-infected adults contributed a median of 4.5 years of followup had shown that the ischemic stroke incidence was 2.26 per 1,000 person-years (95% CI: 1.53, 3.21), approximately 1.5 times the rate of a population-based cohort in North Carolina [6]. 

## 3. Clinical Vignette

Mr. H, a 31-year-old Hispanic male who was admitted to Lincoln Medical and Mental Health Center for acute right MCA territory stroke in view of 5-hour history of sudden onset slurred speech with left upper and lower extremities weakness. There were no history of loss of consciousness, seizures, fever, headache, nausea or vomiting. Review of systems was otherwise unremarkable.

The patient has medical history of HIV infection which was diagnosed 2 years prior to this admission and started on ARTs since then. Mr. H admits compliance to his ARTs but does not remember last CD4 cell count. On the day of admission, CD4 cell count was 32 cells/uL. He was taking Raltegravir 400 mg bid, emtricitabine/tenofovir 1 tablet PO daily, ritonavir 100 mg bid, and darunavir 600 mg bid as well as daily bactrim for pneumocystis (carinii) Jiroveci pneumonia (PCP) prophylaxis. The patient denies cigarettes smoking, alcohol, or substance abuse. 

On examination, the patient was alert and oriented to person, place, and time; the pupils were equal and reactive to light and accommodation. The patient has significant left hemiplegia with strength of 1 : 5 in the left upper and lower extremities; left facial droop was also noted. Neurologic exam was otherwise unremarkable.

His labs showed normal lipid profile (LDL-c of 51 mg/dL and HDL-c of 62 mg/dL), negative Factor V Leiden and prothrombin mutation, normal serum levels of protein C function (222%, normal range: 70–140), protein S function (100%, normal range: 70–142), antithrombin III function (139%, normal range: 75–125), and Homocystein (8.24 umol/L, normal < 12.99). Urine toxicology was negative for cocaine. Serum ANA was negative. Routine CSF analysis was unremarkable and negative for VDRL, Toxoplasmosis Ig M/Ig G serology, and HSV PCR. 

**Figure 1:**
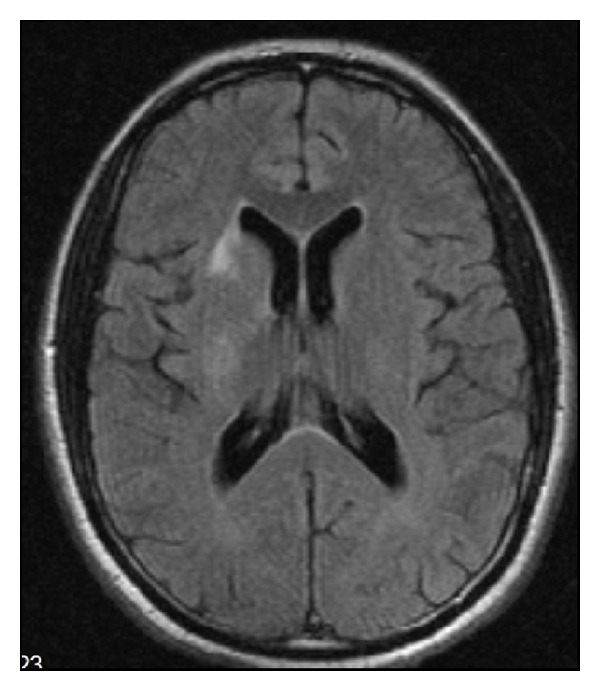
Brian MRI without gadolinium shows right anterior corona radiata and basal ganglia acute infarction.

**Figure 2:**
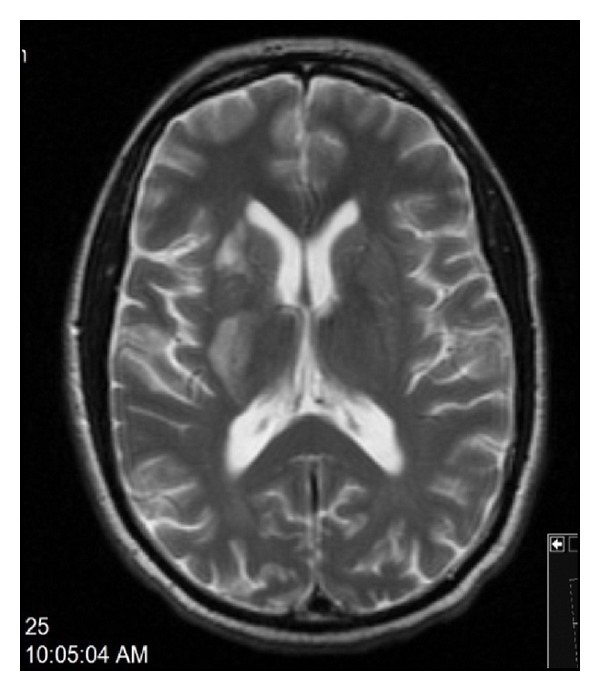
Brain MRI with gadolinium shows the infarct clearly.

Brain imaging studies showed right anterior corona radiata and basal ganglia acute infarction (Figure 1) and more clearly shown on brain MRI with contrast (Figure 2). The patient was started on aspirin and statins as secondary prevention as he was not candidate for thrombolytic therapy. Bilateral vascular Doppler showed normal carotid arteries. Echocardiogram including bubble study was unremarkable. The patient was transferred to skilled nursing facility for rehabilitation. 

## 4. Discussion

Before talking about the risk factors of atherosclerosis in HIV patients, we will talk in brief about the differential diagnosis of stroke-like symptoms in these particular patients. As a common concept, there are two main pathologies cross to our mind in any patient with HIV infection presented with focal neurologic deficits which are the following. 

*(1) Cerebral Toxoplasmosis.* It typically presents with headache, confusion, and fever as well as focal neurological deficit in variant degrees. In one retrospective review of 115 cases, 55, 52, and 47% had headache, confusion, and fever, respectively, and 69% had focal neurologic deficits [7]. Cerebral toxoplasmosis usually affects HIV-infected patients with CD4 count of less than 200 cells per cubic millimeters and not taking toxoplasmosis prophylaxis. 

*(2) CNS Lymphomas.* As the lifespan of HIV-infected patients has increased, malignancies have become a known cause of morbidity and mortality in this population. CNS lymphomas are more common in HIV-infected patients than in the non-HIV-infected patients (about 15 versus 1 percent of all lymphomas). 

A rare culprit for stroke in HIV-infected patients is meningovascular syphilis. Syphilitic meningitis can cause an infectious arteritis that may affect any vessel in the subarachnoid space surrounding the brain or spinal cord and result in thrombosis, ischemia, and infarction. Many patients with meningovascular syphilis have prodromal symptoms, such as headache, dizziness, or personality changes, for days or weeks before the onset of ischemia or stroke. These symptoms are probably due to concomitant meningitis, but those patients may present with typical neurologic deficits leading to the diagnosis of stroke [8]. 

Several causative mechanisms have been proposed for the relationship between HIV infection and cardiovascular risk including HIV-associated dyslipidemia, endothelial dysfunction, inflammation, and hypercoagulability. Let us address these issues and review some of the related studies.

### 4.1. HIV-Associated Dyslipidemia

 HIV-infected patients tend to develop decreases in high-density lipoprotein cholesterol (HDL-c) and low-density lipoprotein cholesterol (LDL-c) levels, followed by an increase in plasma triglyceride levels prior to developing AIDS and the degree of viremia may correlate with the amount of triglyceridemia [9].

A prospective study of 50 men from the Multicenter AIDS Cohort Study has shown significant declines in mean serum total cholesterol (TC), HDL-c, and LDL-c [10], and subsequent initiation of ARTs was associated with increases in TC and LDL-c but little change in HDL-c (it is noteworthy to mention that TC and LDL-C were observed after about 3 years of ARTs which possibly represent a return to pre-infection serum lipid levels after accounting for expected age-related changes). 

A cross-sectional study done in India has shown that of the 306 patients taking ARTs for more than one year (126 controls, 30 on ZDV/3TC/NVP, and 150 on d4T/3TC/NVP), the prevalence of lipodystrophy was 46.1%, and lipoatrophy was significantly associated with d4T use [11]. However, discontinuation of ARTs may increase the risk of death by raising IL-6 and D-dimer levels [12].

### 4.2. Endothelial Dysfunction

 An increased pulse wave velocity (which may be an early marker for atherosclerosis) has been noted in two small studies of HIV-infected patients taking ARTs compared to HIV-seronegative controls [13]. Endothelial function was assessed by brachial artery flow-mediated dilation (FMD) in treatment-naive patients initiating ARTs with either a PIs-based, NNRTIs-based, or nucleoside analog-sparing regimen [14]. Rapid and significant improvement in FMD was seen in all treatment arms compared to baseline, suggesting that suppression of HIV and not the specific drugs themselves led to improved endothelial function [14, 15]. 

However, antiretroviral medications may independently contribute to endothelial damage. In one study, plasma levels for markers of endothelial dysfunction (P-selectin and t-PA) were measured in 60 patients on ARTs and 60 treatment-naïve patients. Significantly higher levels of P-selectin and t-PA levels were found in those taking ARTs compared to the treatment-naïve group [16]. Certain antiretroviral agents may be more likely to disturb endothelial function than others like indinavir, [17] abacavir [18]. 

### 4.3. Hypertension

A Multicenter AIDS Cohort Study followed 5578 HIV-positive participants from 1984 to 2003 and observed a significantly higher systolic blood pressure in those using 8ARTs for greater than five years, [19] and there was no significant increase in systolic blood pressure in which ARTs were used for less than two years. 

### 4.4. Insulin Resistance and Diabetes

ARTs are associated with an increased incidence of insulin resistance and diabetes. A multicenter AIDS cohort study done in Johns Hopkins University has shown that the incidence of DM in HIV-infected men with ARTs exposure was greater than 4 times that of HIV-seronegative men [20]. 

### 4.5. Chronic Inflammation

 HIV infection per se may contribute to cardiovascular disease (CVD) risk via nonspecific inflammation. Uncontrolled HIV infection is associated with elevated markers of inflammation, including CRP; levels of these markers decline with treatment but not to normal levels, [21] and analysis from the FRAM study cohort has shown that fibrinogen and CRP are strong and independent predictors of mortality in HIV-infected adults even in those with relatively preserved CD4 counts >500 cells per microliter [22].

### 4.6. Hypercoagulability

 Despite the endothelial damage and dysfunction discussed above, there is also evidence of an increased predisposition towards a hypercoagulable state. Researchers have described elevated plasma levels of endothelial cell products, including von Willebrand factor (vWF) and soluble thrombomodulin (sTM) in those living with HIV [23]. sTM was strongly raised in those patients with the lowest CD4+ cell count (*P* < 0.001), but levels of vWf increased at each incremental fall in CD4 cell count. A positive correlation has also been noted between HIV viral load and prothrombin fragment in 90 HIV-infected young individuals (mean age 38 ± 9 years, mean CD4 cell count 216 ± 198) not receiving ARTs suggesting that increasing levels of viremia may increase the risk of thrombosis [24]. 

In a study of 49 consecutive patients with acute opportunistic infections were screened for thrombophilic parameters, a followup investigation was performed after 10 ± 8 weeks in 26 patients. In acutely ill patients, the incidence of protein S deficiency was 67% (33/49) and of protein C deficiency 25% (12/49), while at the followup visit the incidences were 54% (14/26) and 8% (2/26), respectively, [25] which still above the national average of protein C and S deficiency. 

By applying the aforementioned information to our case, this young patient without any of the classical risk factors for stroke and had negative thrombophilia workup found to have poorly controlled HIV infection despite being on ARTs for about 2 years. Although the acute thrombosis lowers the levels of protein C, protein S, and antithrombin as well as the initiation of Warfarin lowers the levels of protein C and protein S, normal values rule out those factors as risk factors for hypercoagulability in this patient. A negative VDRL in CSF and the lack of other symptoms of meningitis make the diagnosis of meningovascular syphilis very unlikely. By reviewing the literature, we found that the poorly controlled HIV infection and/or ARTs might be independent risk factors for stroke or acute cardiovascular event. 

## 5. Conclusion

Despite the overall decrease in the number of stroke hospitalizations, data obtained from the Nationwide Inpatient Sample in the United States showed an increase in the number of stroke hospitalizations in the HIV-infected population. 

The introduction of protease inhibitors and nonnucleoside reverse-transcriptase inhibitors for the management of human immunodeficiency virus (HIV) infection dramatically changed the prognosis and survivals, but unexpected cardiovascular events were observed. On the other hand, a poorly controlled HIV infection might be a independent risk factor. 

More studies needed to address this medical dilemma in HIV-infected patients along with other classical risk factors modification which is a must.
